# Strategies and Best Practices for Engaging Urology Residents in Clinical Research during Training

**DOI:** 10.1007/s11934-025-01317-w

**Published:** 2026-01-17

**Authors:** Kate Dwyer, Elisabeth M. Sebesta

**Affiliations:** Department of Urology, Vanderbilt University Medical Center, A1302 Medical Center North, Nashville, TN 37232 USA

**Keywords:** Resident education, Medical education, Research, Mentorship

## Abstract

**Purpose of review:**

In this review, we will explore the benefits of urology resident participation in research during training and identify the common barriers for residents to engage in scholarly activity. Most importantly, we aim to discuss strategies to foster engagement in research during urology residency training despite time constraints, resource limitations, and competing clinical demands.

**Recent findings:**

Recent literature on the topic highlights that while urology residency programs require some form of scholarly activity, few offer extended dedicated research time and there is no minimum requirement for promotion or graduation across programs. Protected research time, structured curricula, and available mentorship are associated with increased research productivity. Encouraging innovative approaches such as longitudinal research exposure and integrated research didactics, incentivization and reward culture, fostering high-quality mentorship, and standardizing expectations have shown some success in improving publication rates and promoting conference presentations.

**Summary:**

The benefits of residents participating in research during training include refined clinical thinking, promotion of evidence-based practice, and facilitating academic career opportunities. We hope to offer a roadmap for programs and educators to consider how to cultivate the culture of inquiry needed to promote resident research engagement without the need to extend training. By starting this conversation, we aim to inspire urology training programs to prioritize research integration in a way that prepares residents to contribute meaningfully to scientific advancement and ultimately improve patient care.

## Introduction

Clinical research is a cornerstone of medical education and a critical component of careers in academic medicine. For trainees, engaging in research during residency training offers opportunities to develop acumen in evidence-based practice, critical thinking, and innovation. However, balancing research with the multitude of demands in residency training remains a significant challenge. Urology residents face competing priorities every day—mastering pathophysiology and anatomy, developing strong clinical decision-making skills, and acquiring surgical proficiency in endoscopic, minimally invasive, and open techniques. These core components form the backbone of surgical training, marshalling time and dedication that in turn limits bandwidth for scholarly activity.

Residency is a formative stage of professional and personal growth. Beyond clinical and technical training, residents are simultaneously responsible for managing career development, academic achievement, and self-promotion for fellowship or faculty positions. These competing demands can create tension, as residents strive to meet clinical expectations, and so often building a scholarly portfolio falls to the wayside in favor of other endeavors. Furthermore, trends in graduate medical education have moved away from adding extra years to training for research or even specialized fellowships, which reduces time for research to take precedent.

Solving this problem within the confines and timeline of residency requires structural change.

The Accreditation Council for Graduate Medical Education (ACGME) Program Requirements for Graduate Medical Education in Urology state, “It is recognized that programs may place different emphasis on research, leadership, public health, etc. program aims will reflect the nuanced program specific goals.” The requirements go on to state generally “The program must advance residents’ knowledge of the basic principles of research, including how research is conducted, evaluated, explained to patients, and applied to patient care.” Likewise, faculty in a program, must participate in scholarship in at least 3 of 7 domains^1^[1]. Given this lack of specificity in requirements, research experience and exposure during urology residency are highly variable across programs. Some programs offer structured research curricula and dedicated research time, while others provide minimal formal support. Less than 20% of programs have extended dedicated research time [2]. This inconsistency results in markedly different experiences for trainees, and influences their opportunities to publish, present, and maybe even pursue fellowship or academic careers in the future. Moving forward, programs should take an intentional and innovative approach to integrate research within the existing confines and timelines of urology training to facilitate broader accessibility and maintain academic achievement as a cornerstone of medical education.

**Fig. 1:**
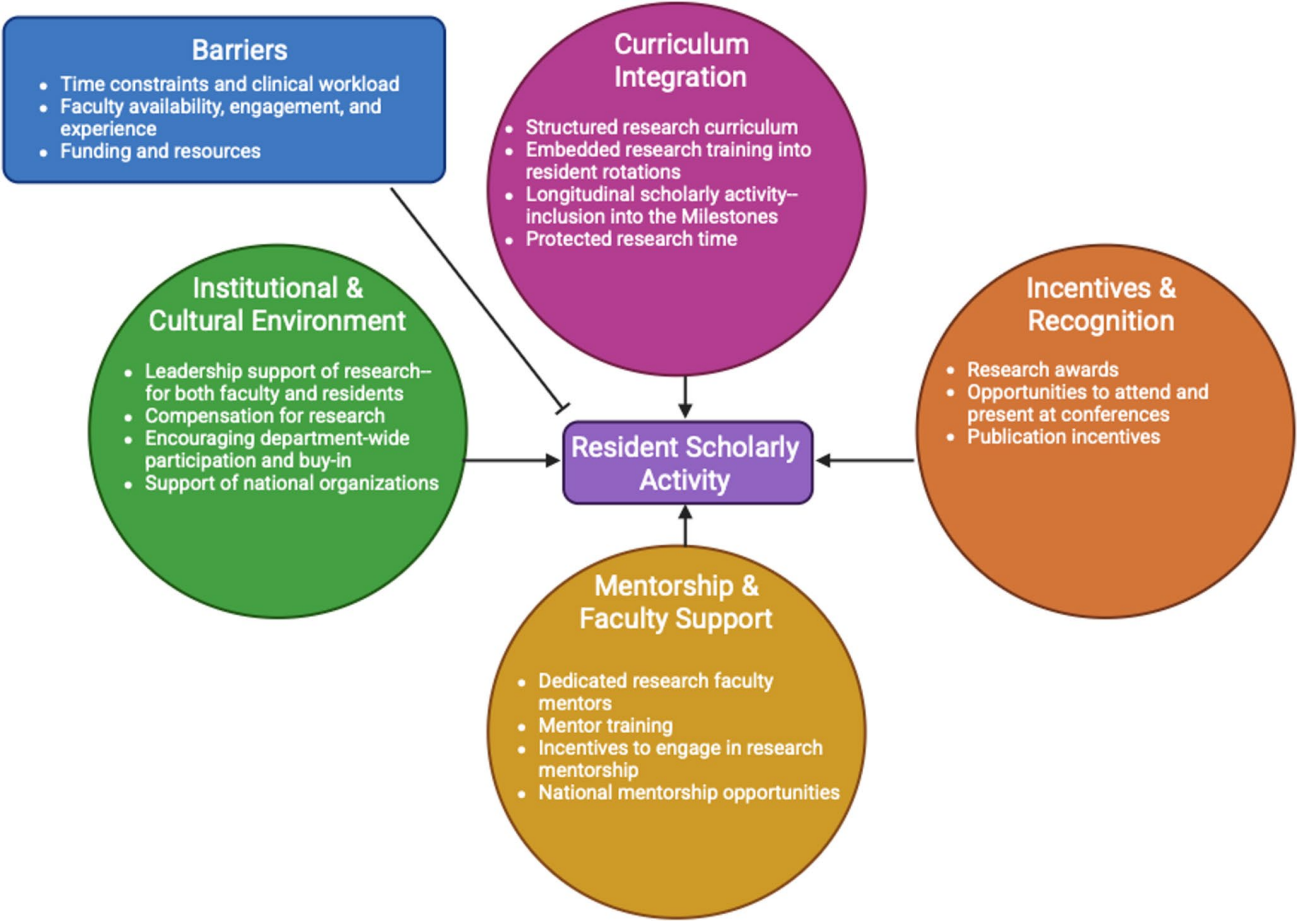
Conceptual model for encouraging resident scholarly activity (Created in BioRender. Sebesta, E. (2025) https://BioRender.com/337hbkl)

In this review, we will examine the barriers that limit resident participation, explore the benefits of engaging in research and scholarly activity during urology residency training, and propose strategies and best practices to foster research engagement. See Fig. 1 for a summative conceptual model. Ultimately, promoting research engagement among urology residents is essential for advancing the field, improving patient care, and preparing the next generation of academic leaders in the field. Therefore, by addressing structural, cultural, and resource challenges, urology programs can create environments that support scholarly activity without compromising clinical training.

## Barriers Throughout the Research Process in Residency Training

While the importance of research in medical training is widely acknowledged by educators and residents alike, hurdles throughout the research pipeline continue to limit meaningful engagement in scholarly activity during residency. In order to ensure research remains an integral part of urology training, it is essential to recognize the roadblocks at each step.

### Barriers to Initiating a Project

Developing a clinically relevant and answerable question requires a strong grasp of the existing literature and an inquiring mind. Mentorship is a key facet of developing this skill. Residency programs that lack faculty actively engaged in research with the experience and availability to mentor trainees cannot reasonably expect their residents to produce meaningful scholarly work.

A higher proportion of faculty participating in research has been associated with increased resident presentations at academic meetings [3]. Additionally, dedicated research directors, protected faculty research time, and chairperson support for scholarly activity further increase resident research productivity [3]. Likewise, formal mentorship programs have also been shown to increase resident publication rates [4].

The absence of a formal research curriculum for residents has also been associated with decreased productivity [3, 5]. Residents may struggle to understand program requirements, which can make it difficult to identify realistic projects. Along with faculty engagement, deliberate efforts to establish formal mentorship and curriculum frameworks are beneficial to fostering resident research success.

Lastly, money remains another significant barrier. Research during residency often relies on departmental funds, from clinical revenue or endowments [2]. Only 39% of urology programs report providing full funding for resident research, while 24% report absolutely no funding [3].

### Barriers to Conducting Research

Even after a project has been started, it takes time, access to data, coordination, and statistical support. There has been a trend over the past two decades towards a shorter duration of medical training with the goal of minimizing delayed income. At the same time, in 2003, the ACGME limited resident duty hours to an 80-hour week. Time may be the most limiting factor for pushing a project to completion.

While resident-focused interventions such as peer-review training address important skill gaps in an expedited fashion, they do not overcome structural constraints within residency programs [6].

While 98% of urology residency programs report requiring some form of scholarly activity for graduation, fewer than 20% offer extended, dedicated research blocks, and most research time is integrated into clinical rotations [2, 3]. In 2024, only 17 of 143 urology residencies in the United States include a 6th year [6]. Residents themselves do seem to value research exposure during training. In a survey of trainees, over 70% reported research enhanced their education and training [7]. However, only 30% stated they would be willing to complete a 6-year training program with a dedicated research year.

Without dedicated research time, there tends to be rapid erosion of the time available for research for clinical responsibilities [2]. Lack of time has consistently emerged in the literature as a significant barrier for residents engaging in research during training. Increased time dedicated to research during training is directly proportional to greater research productivity [8]. 6-year programs with a dedicated research year have significantly greater scholarly output as compared to 5-year programs [9, 10]. Dedicated time has been linked to increased participation in research, publications, and scholarly presentation [3]. Yet the trend towards shorter training throughout medicine persists. This means that fewer and fewer urology programs offer this dedicated research time, and fewer trainees seek this additional time during training. This tension is one of the major barriers to overcome: resident research is valued and important for future career success but in modern urology training there is decreasing time dedicated to research.

### Barriers to Completing and Disseminating Work

Essential resources to complete high-quality research, such as travel funds to academic conferences, IRB and statistical support, and dedicated research directors all require financial investment and have been associated with improved scholarly activity [3]. One study looking at anesthesiology residents found that the cost to the department for a dedicated research resident was $13,500 per month, with additional costs for travel to conferences and time away from clinical duties, which is prohibitive for some departments [11]. Additionally, the current climate of increasing constraints on research funding leads to fewer opportunities for research experience, publications, and exposure to research during urology training. Navigating this landscape with new and innovative strategies will be critical to continue to encourage urology resident research engagement.

There are numerous other barriers that residents face when it comes to prioritizing research during training. This can include limited physical space, administrative hurdles and lack of support to navigate, institutional culture which prioritizes clinical activities over research, lack of perceived benefits by residents, among others. Programs that fail to integrate research into the core mission of their training may struggle to create an environment which is conducive of inquiry and innovation during residency.

## The Value of Resident Research Involvement and the Case for Reform

Despite these shifting attitudes and challenges, research during residency offers significant career benefits that we can lose sight of with the multiple competing priorities during training. Increased dedicated research time and publication output during residency have been associated with greater academic productivity in early career urologists and obtaining NIH funding and professorships later on [8, 12]. Similarly, residents who complete research and publish more frequently are more likely to pursue a career in academics over private practice [13].

While access to research during residency correlates with academic advancement, a minority of practicing urologists actually work in academic practice. In the 2024 American Urological Association (AUA) Census, only about 30% of practicing urologists worked in an academic-affiliated medical center [14]. Nevertheless, research engagement remains critical for all clinicians to stay current with emerging evidence throughout their career. Hospitals with higher levels of scholarly activity have been linked to improved patient outcomes, including lower mortality rates [15]. This suggests that involvement in research promotes rapid integration of new evidence, better implementation of evidence-based practice, and more informed clinical decision-making. Furthermore, research fosters a culture of inquiry—institutions that actively engage in research encourage continuous learning and adaptive clinical practice, supporting quality improvement [16].

For the 70% of urologists who work outside of academic environments, skills in research and inquiry can be highly valuable for quality improvement (QI) and patient safety. As mentioned above, ACGME includes a requirement for teaching QI methods and implementation. In addition, the American Urological Association has developed the program Engage with Quality Improvement and Patient Safety (E-QIPS) to generate interest and provide support [17]. These efforts are universally applicable including topics like increasing clinical efficiency, minimizing opioids, or improving bladder cancer surveillance.

Regardless of a trainees’ targeted practice setting for their careers, developing an understanding of research methodology, critical evaluation of the literature, and evidence integration into practice begins during residency. Therefore, it is essential to cultivate these skills in residency to promote future contributions to the field of urology and to advance patient care.

## Strategies To Foster Engagement

### Curriculum Integration

#### Embedding Research Training into Residency Programs

One of the most critical components for promoting resident engagement in research is actively embedding research training into the residency program. As discussed above, residents are increasing reluctant to pursue additional years of training, therefore, we cannot rely on dedicated research years during residency or even fellowship for research experience as we once did. Therefore, programs must find innovative and creative ways to integrate research into the traditional 5-year urology curriculum without compromising core clinical training or adding excessive work outside of clinical time which may contribute to burnout. While this may seem challenging, it is feasible and important.

One possible approach is the development of a longitudinal research curriculum. Rather than adding extra years to training, programs can consider implementing structured research education which spans the entirety of training. This might include incorporating research seminars, biostatistics classes, and lessons in peer review into existing didactic schedules. Additionally, journal clubs can serve as a platform to teach crucial skills in critical appraisal of the literature [6].

In other medical specialties, this has been done well. Numerous publications suggest that a formal research curriculum increases resident scholarly productivity [18]. In emergency medicine, implementing a formal longitudinal research program with milestones throughout training lead to higher publication rates, more presentations, and increase in first-author manuscripts by the end of training [19]. This program included a didactic curriculum, specific benchmarks, research funding, statistical support, and a dedicated research coordinator. While a program this comprehensive may be cost-prohibitive for many departments, adopting some key elements—including structured timelines, integrated teaching, and mentorship models—could be used to substantially enhance research engagement. Making research part of the schedule and not an afterthought is key.

Finally, to enhance accountability and standardization, it is worth considering whether research could be formally integrated into the Milestones and be linked to resident promotion throughout training. Although the ACGME requires that residents must participate in scholarly activity, current urology Milestones do not specify research-related benchmarks linked to resident promotion. Linking advancement to tangible research output is one way to encourage consistent engagement and skill development—such as requiring proposal submission, data collection, presentations at conferences, and/or manuscript submission by certain levels of training.

#### Protected Research Time

Protected time to conduct research is critical for fostering residency scholarly activity. While full research years significantly increase productivity, even limited protected time embedded within existing rotations or clinical schedules has the potential to substantially improve research engagement and output. In Lee et al.’s study of 36 urology programs, the authors were able to categorize duration of research experience into 3–4 months, 6 months, and 12 months effectively demonstrating a significant dose-response with increasing productivity given more time and confirming that even incremental change can make a difference [12].

Programs should plan rotation schedules with research in mind. Chhor et al. described this in radiology residency, implementing one half-day of research per week as part of existing rotations [20]. Authors demonstrated this lead to a 14% increase in abstract presentations as well as an impressive 67% increase in publications and 275% increase in textbook chapters. The increase in conference presentations was more subtle, perhaps because that was a more attainable goal prior to the addition of this designated time. However, authors note that apart from a pure numeric increase, research engagement in this longitudinal fashion resulted in deeper engagement and more robust academic output in the form of first author publications and book chapters. These findings align with other literature as well. For example, a 2025 study across multiple specialties found that residents may seek out systematic review and observational studies, traditionally considered to be of lower academical rigor but more reasonable goals in truncated timeframes [21].

Rather than expecting residents to complete research during nights or weekends—an approach that risks burnout and limits resident buy-in—programs should integrate research time into the clinical schedule. Although a short block alone may not allow completion of a major research project, we do not need to expect urology residents to run clinical trials or do strictly bench research to have a meaningful research experience in training. When combined with a longitudinal curriculum, these intervals can propel projects towards deepened engagement and productivity. Finally, it is ultimately up to the faculty educators and program leadership to advocate for this integration; without deliberate design of training programs and advocating for protected time, expectations for resident scholarly productivity are unrealistic.

### Mentorship and Faculty Support

Adequate faculty mentorship is essential for resident research success. Programs must have faculty who are activity engaged in research themselves, possess relevant expertise, and are available and willing to provide guidance to trainees. Beyond its role in reducing burnout, improving career satisfaction, and supporting diversity, mentorship can directly influence research productivity [22]. Faculty mentorship for research is a critical component for identification of research opportunities, research productivity, and cultivating future interest in pursuing academic careers. However, barriers to this also exist. One recent study of practicing urologists to assess the factors or barriers that encourage or limit faculty research mentorship, 20% reported never being a research mentor, with lack of sufficient time and lack of institutional support limiting faculty’s ability to provide research mentorship to residents [23]. Departments can help mitigate these challenges by incentivizing mentorship to residents—whether through RVU compensation, bonus pay, or compensating or protecting time for research mentorship. Likewise, departments may consider providing mentorship training to faculty to encourage effective mentor-mentee relationships. Finally, having a faculty point person, a research director, can also be effective. Having one person to help oversee all resident productivity can help keep everyone on track.

For programs who do not have faculty who are experienced in research available and/or willing to provide mentorship, there are numerous multi-institutional efforts in urology to help foster mentorship relationships across institutions. These include UnderRepresented Trainees Entering Residency (UReTER) program, societal mentorship programs through Society of Women in Urology (SWIU) and the R. Frank Jones Urological Society, and efforts through the AUA via leadership and mentorship programs. If inadequate support for research exists, program leadership should aim to connect residents with external research mentors.

### Incentives and Recognition

The benefits of engaging in research can be less tangible than other aspects of residency training. For example, in surgical skills acquisition the progress may be immediately visible—by doing more challenging cases and gaining increased autonomy. The benefits of research, however, are often more abstract and delayed, without immediate gratification felt in the same way.

Even with time, curriculum, and mentorship in place, incentives can be an effective measure to increase resident research engagement. Monetary rewards for publications have been shown to increase productivity [24]. Other incentives can also be considered to provide tangible motivation—departmental funding for conference travel, academic awards, rewards, or time off. Therefore, offering an immediate recognition and rewards can bridge this gap and sustain resident commitment to scholarly activity.

### Institutional and Cultural Environment

Finally, a strong institutional and departmental culture of fostering innovation and inquiry is essential. Departmental leadership must prioritize research as a core component of training, not an optional endeavor. Faculty—whether active in research themselves or not—should support the department’s initiatives that allow residents protected time for scholarly work. National organizations in urology, such as the AUA and the Society of Academic Urologists (SAU) promote research during training, offer mentorship and leadership opportunities, and promote funding for trainees; however, it is important for residents to see these same values reinforced at the local leadership level. As discussed above, integrating a research curriculum into departmental didactics is an excellent way to involve all faculty in the department in scholarly initiatives, and help promote a culture of inquiry and innovation. Holding research forums to highlight the research being conducted within the department or having a departmental poster contest after residents present at national meetings can all help to highlight resident research initiatives and create a positive culture around research.

## Future Directions

As we look towards the future of urology residency training and the integration of research and scholarly activity, several key considerations emerge.

Virtual platforms offer a powerful solution to help overcome geographic and institutional disparities for residents to engage in research that interests them. Not all programs have equal resources, but our trainees should not be disadvantaged because of this. Virtual mentorship can connect residents from programs with limited research faculty to mentors at institutions with more robust research infrastructure. Similarly, research curricula or didactic sessions can be delivered virtually through live webinars or recorded modules accessible across regions or nationally, mitigating the need for local experts to teach research didactics. The expansion of virtual education is a lasting benefit from the COVID-19 pandemic, and resident research training should follow suit.

As mentioned above, another future direction could be integration of scholarly activity into the ACGME Milestones to standardize expectations across programs. Clear guidelines on minimum research requirements for promotion and graduation would left ensure consistency in urology training. Likewise, establishing a baseline policy for protected research time across programs could guarantee equitable opportunities for all trainees. Additionally, this could allow for standardized reporting of resident scholarly activity for program accreditation, which would further promote accountability and resource allocation.

## Conclusion

Research during training is vital. The benefits extend beyond academic achievement to improved patient care and lifelong integration of evidence-based practice. Yet, there are significant barriers—time constraints, limited resources, and lack of mentorship—that continue to hinder urology resident research engagement. We hope through this review we have highlighted the importance of continuing to evolve how we train urology residents to ensure research and scholarly activity remain a priority, and what we can look to for the future of urology residency training.

While long-term changes may take time, we should ask what can we do now as educators, mentors, and urology program leadership? We encourage programs to implement a structured research curriculum and schedule dedicated time for scholarly work—whether through a dedicated block of time or a longitudinal integration—avoid placing the burden entirely on residents’ personal time off clinical duties. Creative solutions to integrate research into the traditional 5-year training timeline is paramount to balance research with clinical training. Consider incentives for both residents and faculty, such as awards, travel support, or even local recognition for efforts. These strategies foster a culture of inquiry and innovation that sustains research engagement.

Ultimately, the future of research in urology training depends on collaboration between national organizations, institutional leadership, and individual programs. By prioritizing research integration and leveraging technology, we can ensure that scholarly activity remains a vital component of resident education and continues to advance our field.

## Key References

- Faber LS, Jurado M, Bennett-Perez R, Alba FM. Scholarly Activity and Research Training in Urology Residency Programs: Assessment of Current Practice and Barriers. Urology. 2022;168:41–9. doi: 10.1016/j.urology.2022.07.011.
  - ○ A recent survey-based study of urology residency programs to analyze the current state of research training and scholarly activity in urology residency training programs which which factors promote and inhibit productivity.

## Data Availability

No datasets were generated or analysed during the current study.

## Footnotes

1 (1) Research in basic science, education, translational science, patient care, or population health (2) Peer-reviewed grants (3) Quality improvement and/or patient safety initiatives (4) Systematic reviews, meta-analyses, review articles, chapters in medical textbooks, or case reports (5) Creation of curricula, evaluation tools, didactic educational activities, or electronic educational materials (6) Contribution to professional committees, educational organizations, or editorial boards (7) Innovations in education.

## References

[1] Accreditation Council for Graduate Medical Education (ACGME). Program requirements for graduate medical education in Urology. https://www.acgme.org/globalassets/pfassets/programrequirements/2025-reformatted-requirements/480_urology_2025_reformatted.pdf (2025). Accessed December 16, 2025.

[2] Montie J, Faerber G, Schaeffer A, Steers W, Liebert M, Stoll D, et al. Urology residency and research: round table discussion and plea for innovation. Urology. 2008;71(5):762–5. 10.1016/j.urology.2007.10.040. 18295864 10.1016/j.urology.2007.10.040

[3] Faber LS, Jurado M, Bennett-Perez R, Alba FM. Scholarly activity and research training in urology residency programs: assessment of current practice and barriers. Urology. 2022;168:41–9. 10.1016/j.urology.2022.07.011. 35882304 10.1016/j.urology.2022.07.011

[4] Wood W, McCollum J, Kukreja P, Vetter IL, Morgan CJ, Hossein Zadeh Maleki A, et al. Graduate medical education scholarly activities initiatives: a systematic review and meta-analysis. BMC Med Educ. 2018;18(1):318. 10.1186/s12909-018-1407-8. 30577779 10.1186/s12909-018-1407-8 PMC6303993

[5] Gangwish DJ, Parshall CA, Qeadan F, Jurado M, Bennett RN, Alba FM. Predictors and barriers to faculty scholarly activity in united States urology residency programs. Urology. 2020;139:37–43. 10.1016/j.urology.2019.10.047. 31991142 10.1016/j.urology.2019.10.047

[6] Dwyer K, Koch GE. Unlocking peer review: elevating scholarly writing and research competence in urology residency. Curr Urol Rep. 2024;25(7):163–8. 10.1007/s11934-024-01208-6. 38836977 10.1007/s11934-024-01208-6

[7] Peyton CC, Badlani GH. Dedicated research time in urology residency: current status. Urology. 2014;83(4):719–24. 10.1016/j.urology.2013.09.072. 24508251 10.1016/j.urology.2013.09.072

[8] Yang G, Zaid UB, Erickson BA, Blaschko SD, Carroll PR, Breyer BN. Urology resident publication output and its relationship to future academic achievement. J Urol. 2011;185(2):642–6. 10.1016/j.juro.2010.09.097. 21168863 10.1016/j.juro.2010.09.097 PMC3565588

[9] Finkelstein JB, Van Batavia JP, Rosoff JS. The difference a year can make: academic productivity of residents in 5 vs 6-year urology programs. Urology. 2015;86(2):220–2. 10.1016/j.urology.2015.03.057. 26209453 10.1016/j.urology.2015.03.057

[10] Thompson RH, Lohse CM, Husmann DA, Leibovich BC, Gettman MT. Predictors of a successful urology resident using medical student application materials. Urology. 2017;108:22–8. 10.1016/j.urology.2017.06.046. 28751165 10.1016/j.urology.2017.06.046

[11] Schott NJ, Emerick TD, Metro DG, Sakai T. The cost of resident scholarly activity and its effect on resident clinical experience. Anesth Analg. 2013;117(5):1211–6. 10.1213/ANE.0b013e3182a44d5d. 24108257 10.1213/ANE.0b013e3182a44d5d

[12] Lee A, Namiri N, Rios N, Enriquez A, Hampson LA, Pruthi RS, et al. Dedicated residency research time and its relationship to urologic career academic success. Urology. 2021;148:64–9. 10.1016/j.urology.2020.10.037. 33166543 10.1016/j.urology.2020.10.037

[13] Warren CJ, Voleti SS, Stevens V, Brown SJ, Schroeder M, Yee C, et al. PubMed-indexed research productivity of urology applicants and residents: does medical student research productivity predict resident research or pursuit of an academic career? Urology. 2025;195:43–9. 10.1016/j.urology.2024.10.001. 39395447 10.1016/j.urology.2024.10.001

[14] American Urological Association Education and Research IA. The state of the urology workforce and practice in the united states 2024. Maryland: Linthicum; 2024.

[15] Hanney SR, Gonzalez-Block MA. Organising health research systems as a key to improving health: the World Health Report 2013 and how to make further progress. Health Res Policy Syst. 2013;11:47. 10.1186/1478-4505-11-47. 24341347 10.1186/1478-4505-11-47 PMC3878484

[16] Boaz A, Hanney S, Jones T, Soper B. Does the engagement of clinicians and organisations in research improve healthcare performance: a three-stage review. BMJ Open. 2015;5(12):e009415. 10.1136/bmjopen-2015-009415. 26656023 10.1136/bmjopen-2015-009415 PMC4680006

[17] American Urological Association (AUA). Engage with quality improvement and patient safety (E-QIPS). https://www.auanet.org/guidelines-and-quality/quality-and-measurement/quality-improvement/engage-with-quality-improvement-and-patient-safety-(e-qips) Accessed December 16, 2025.

[18] Lennon RP, Fuentes RWC, Broszko C, Koch JJ, Sanchack K, Keck JW. A curriculum to increase resident scholarly activity. Fam Med. 2020;52(8):557–61. 10.22454/FamMed.2020.257274. 32672834 10.22454/FamMed.2020.257274

[19] Krispin S, Kontowicz E, Faine B, Takacs M, Harland KK, Vakkalanka JP, et al. Resident scholarly activity and productivity outcomes before and after implementing a structured research program: a before-after study. AEM Educ Train. 2025;9(4):e70082. 10.1002/aet2.70082. 40718520 10.1002/aet2.70082 PMC12286887

[20] Chhor CM, Fefferman NR, Clayton PM, Mercado CL. Impact of longitudinal focused academic time on resident scholarly activity. Acad Radiol. 2022;29(12):1903–8. 10.1016/j.acra.2022.02.025. 35361538 10.1016/j.acra.2022.02.025

[21] O’Shea M, Ashcherkin N, Arunachalam Karikalan S, Biondi M, Chaffin H, Umar S, et al. A mixed methods survey of research education requirements for residents in internal medicine, neurology and transitional programs. Med Educ Online. 2025;30(1):2494579. 10.1080/10872981.2025.2494579. 40271579 10.1080/10872981.2025.2494579 PMC12024497

[22] Chen A, Harnett J, Kothari P, Ernst M. A review of mentorship in urology: are we satisfied? Curr Urol Rep. 2022;23(12):383–92. 10.1007/s11934-022-01122-9. 36459377 10.1007/s11934-022-01122-9 PMC9716155

[23] Horning P, Payne J, Posid T, Palettas M, Lehman A, Rose J et al. Urology faculty perspectives on research mentorship. North Central Section of the AUA 94th Annual Virtual Meeting2020.

[24] Larsen RG, Bowdino CS, Van Leeuwen BJ, LaGrange CA, Deibert CM. The positive effect of monetary incentive on urology resident research. Urology. 2020;146:43–8. 10.1016/j.urology.2020.07.077. 32976919 10.1016/j.urology.2020.07.077

